# PCR-based zebrafish model for personalised medicine in head and neck cancer

**DOI:** 10.1186/s12967-019-1985-1

**Published:** 2019-07-22

**Authors:** Ahmed Al-Samadi, Katja Tuomainen, Anne Kivimäki, Abdelhakim Salem, Sakhr Al-Kubati, Aini Hyytiäinen, Mataleena Parikka, Karri Mesimäki, Tommy Wilkman, Antti Mäkitie, Reidar Grenman, Tuula Salo

**Affiliations:** 1 Department of Oral and Maxillofacial Diseases, Clinicum, Faculty of Medicine, University of Helsinki, Biomedicum Helsinki 1, C223b (Haartmaninkatu 8), P.O. Box 63, 00014 Helsinki, Finland; 2 Faculty of Medicine and Health Technology, Tampere University, Tampere, Finland; 3 Oral and Maxillofacial Unit, Tampere University Hospital, Tampere, Finland; 4 Department of Oral and Maxillofacial Surgery, HUS Helsinki University Hospital, Helsinki, Finland; 5 Department of Otorhinolaryngology – Head and Neck Surgery, HUS Helsinki University Hospital and University of Helsinki, Helsinki, Finland; 6 Division of Ear, Nose and Throat Diseases, Department of Clinical Sciences, Intervention and Technology, Karolinska Institutet, Karolinska University Hospital, Stockholm, Sweden; 7 Research Program in Systems Oncology, Faculty of Medicine, University of Helsinki, Helsinki, Finland; 8 Department of Otorhinolaryngology – Head and Neck Surgery, Turku University Hospital, University of Turku, Turku, Finland; 9 Cancer and Translational Medicine Research Unit, University of Oulu, Oulu, Finland; 10 Medical Research Centre, Oulu University Hospital, Oulu, Finland; 11 Helsinki University Hospital, Helsinki, Finland

**Keywords:** Drug screening, In vivo, Oral cancer, Model, Xenograft, Chemotherapy

## Abstract

**Background:**

Currently, in vivo model for personalised cancer drug testing is challenging. A zebrafish larvae xenograft model has been applied in recent years to cancer research, particularly for drug testing purposes, showing promising results in drug testing against patient-derived tumour xenografts. Currently, these xenograft models apply imaging techniques to measure drug efficacy. However, this method carries several limitations, including timely imaging, thereby reducing the available number of tested fish and drugs. Here, we propose a PCR-based fast assay to evaluate drug efficacy in a zebrafish larvae xenograft model.

**Methods:**

We tested two primary and corresponding metastatic head and neck squamous cell carcinoma (HNSCC) cell lines and patient-derived tongue cancer sample applying zebrafish larvae xenograft model. Cisplatin efficacy was tested using imaging technique and compared the results with PCR-based methods. Drug screening of eight compounds was applied on both cell lines and patient sample using PCR.

**Results:**

In a head-to-head comparison, all the three techniques (imaging, quantitative PCR, and droplet digital PCR) showed similar reduction of the cancer cells growth after cisplatin treatment. Using the quantitative PCR assay, we demonstrated a dose-dependent response of HNSCC cells to cisplatin. Drug screening results of four HNSCC cell lines and patient sample revealed different drug efficacy between tested cancer cells.

**Conclusion:**

We introduce a novel, easy, fast and cost-effective PCR-based in vivo zebrafish larvae assay to test the response of cell lines and clinical tumour samples to anti-cancer drugs. This method goes hand-by-hand with the commonly used imaging assay.

**Electronic supplementary material:**

The online version of this article (10.1186/s12967-019-1985-1) contains supplementary material, which is available to authorized users.

## Background

Head and neck squamous cell carcinoma (HNSCC) is globally the eighth most common malignancy [1], characterised by early metastasis and poor survival. Currently, primary treatment of HNSCC patients consists of surgery and (chemo)radiotherapy either alone or in combination [2]. Other approaches, such as targeted- and immunotherapy, also represent approved modalities, although they are not consistently applied as a first-line approach to treat HNSCC patients. However, these treatments still offer limited efficacy given that 5-year survival amongst HNSCC patients is around 50% [3]. Once the tumour becomes resistant to radio- and chemotherapy, patients may receive various adjuvant treatments, such as cetuximab and pembrolizumab, to improve their survival [4–6]. This strategy leads to several problems including unnecessary side effects, high costs and ineffective treatments. Therefore, personalised treatment of cancer patients in general and HNSCC patients in particular remains a necessity. Unfortunately, until now no practical in vivo system existed for testing cancer-drug efficacy in a patient sample.

A zebrafish larvae xenograft has been used in recent years as a promising in vivo model in cancer research [7–12]. This model carries several advantages, including a large number of offspring, a small size (can fit in a 96-well plate), a short experimental duration, a low cost and the possibility of completing high-throughput testing. All of these factors encourage researchers to shift towards using zebrafish larvae in their experiments. Until recently, the zebrafish larvae model has primarily been used to study cancer cell proliferation, metastasis, tumour angiogenesis and drug testing [9, 13, 14]. Interestingly, two studies reported its application as a model for personalised colon and gastric cancer drug testing using patient-derived xenografts [7, 9]. Despite these positive findings, some drawbacks continue to surround this model, including the evaluation technique of the drug response. Currently, imaging is the only available method for evaluating the tumour size and metastasis. This method is time-consuming and poses limits on the number of fish per test, rendering it difficult for use in high-throughput screening. Currently, all of the published reports about drug testing in zebrafish larvae are limited to three or four drugs. To address this problem, here, we introduce an easy, fast and low-cost PCR-based assay to evaluate the response of cancer cell lines and patient-derived xenografts to anti-cancer drugs. We also compare the results of this assay with an already-established imaging assay.

## Methods

### Cell lines

In this study, we used two primary and two metastatic HNSCC cell lines (kindly provided by Dr. Reidar Grenman’s lab, University of Turku, Additional file 1: Table S1). Cells were cultured in 75 cm^2^ flasks containing Dulbecco’s modified Eagle’s medium (DMEM)/F-12 (Gibco, Paisley, UK) supplied with 10% heat-inactivated foetal bovine serum (FBS), 100 U/ml penicillin, 100 μg/ml streptomycin, 250 ng/ml fungizone and 50 μg/ml ascorbic acid. All cell lines were mycoplasma-free, tested using the PCR Mycoplasma Test Kit I/C (PromoKine, Heidelberg, Germany). To prepare the cell suspensions for injection, cells were detached from the flask using trypsin/EDTA and suspended in media at a concentration of 5 × 10^5^/µl. For imaging purposes, cells were stained using CellTrace Far Red (Invitrogen, Carlsbad, CA, USA) according to the manufacturer’s instructions.

### Patient sample

Our institutional Research Ethics Board (14.03.2016 Eettmk 84) approved this study setting. Patient participation was voluntary and required informed consent. The patient had metastatic oral tongue cancer (Additional file 1: Table S1). After the surgical removal of the tumour and the metastatic lymph nodes, the patient received chemoradiotherapy (66/2 Gy for the primary tumour area + 50/2 Gy effective dose for the lymph nodes and cisplatin infusion 40 mg/m^2^ weekly for 6 weeks). After 3 months, a CT scan showed no signs of recurrence or metastasis.

A fresh tissue sample was obtained perioperatively and placed in a 50-ml falcon tube containing ice-cold Hanks’ Balanced Salt solution (HBSS; supplied with 100 U/ml penicillin, 100 μg/ml streptomycin and 250 ng/ml fungizone). Tissue samples were stored on ice until further processing. Each sample was placed in a petri dish and kept on ice containing HBSS. Necrotic tissues were removed using a scalpel. Vital tissue pieces were placed into a new petri dish containing HBSS and minced into small (1–2 mm) pieces with a scalpel. The tissue pieces were transferred to 15-ml falcon tube and centrifuged for 5 min at 1000 rpm (200×*g*) at 4 °C. After centrifugation, the supernatant was discarded and a fresh HBSS buffer was added before another round of centrifugation. The supernatant was discarded, and the tissue piece pellet was suspended in a 5-ml HBSS buffer containing 1 mg/ml collagenase type I from Clostridium histolyticum (Sigma-Aldrich, St. Louis, Mo, USA) and placed on a rocker platform at 37 °C. After 2 h of incubation, the tube was centrifuged and the supernatant was discarded and replaced with a fresh HBSS buffer before another round of centrifugation. The digested sample was suspended in an HBSS buffer, filtered using a 100-μm cell strainer (Falcon™ Cell Strainer, Fisher Scientific, NH, USA) and the flow-through (single cells) was collected and centrifuged. The supernatant was discarded and the cell pellet was suspended in DMEM/F-12 at a concentration of 5 × 10^5^ cells/µl.

### Zebrafish larvae microinjection and drug administration

Experiments were done at the Zebrafish Unit at the University of Helsinki under the ethical permission (ESAVI/13139/04.10.05/2017) given by the regional state administrative agency. Wild-type zebrafish from the AB strain were maintained as described previously [15] in laboratory fish multi-rack system. The larvae were grown at 28.5 °C in an embryonic medium (5 mM NaCl, 0.17 mM KCl, 0.33 mM CaCl_2_ and 0.33 mM MgSO_4_; Sigma-Aldrich). Two-day-old fish were dechorionated, anesthetised with 0.04% Tricaine and 2 nl of a cell suspension (1000 cells) was microinjected into the perivitelline space. The larvae were transferred to a fresh embryonic medium in a 24-well plate and kept at 34 °C for 72 h and then collected for RNA isolation or fixed for imaging. The zebrafish larvae were treated with 1-phenyl 2-thiourea (PTU) for imaging to avoid pigmentation. We chose eight drugs (one chemotherapy agent and seven targeted-therapy agents) for this assay. Drug concentrations were chosen based on our previous in vitro drug testing as well as on the cytotoxicity test for the zebrafish larvae (Additional file 1: Table S2). Drugs were diluted in the embryonic medium and DMSO was used as the negative control. Fish were kept in 24-well plates (5 fish per well in 1 ml of an embryonic medium). Twenty fish were used for each drug, where ten fish were pooled together to provide a sufficient signal during PCR amplification. The method is in compliance with the ARRIVE guidelines.

### Imaging of the xenograft

Fish were imaged using a Zeiss Axio Imager (Carl Zeiss AG, Oberkochen, Germany) and a Leica TCS SP8 MP CARS (Leica Microsystems, Wetzlar, Germany; Additional file 2: Video S1), and the tumour area was measured using Matlab (MathWorks, Natick, MA, USA).

### Quantitative and droplet digital PCR

RNA was extracted from the fish using the RNeasy Mini Kit (Qiagen, Düsseldorf, Germany) according to the manufacturer’s instructions. In total, 400 ng of RNA were used for the cDNA synthesis using an iScript cDNA synthesis kit (Bio-Rad, Hercules, CA, USA). For quantitative PCR, 10-μl iQ SYBR green, 7-μl water and 1 μl of 250 nM of a primer solution were added to 2 μl of a cDNA sample. Zebrafish Glyceraldehyde 3-phosphate dehydrogenase (GAPDH) was used as the housekeeping gene.

For droplet digital PCR (ddPCR), 10 μl of QX200™ EvaGreen^®^ ddPCR™ Supermix (Bio-Rad Laboratories) and 1 μl of 900 nM of a primer were added to 2 μl of a cDNA sample. Samples, in addition to the Droplet-Generation Oil for EvaGreen (70 μl), were loaded into DG8 cartridges and placed into a QX200™ Droplet-Generator (Bio-Rad Laboratories) for individual droplet generation. Droplets (40 μl) were then transferred to a 96-well PCR plate, sealed with the supplied foil in a PX1-PCR Plate Sealer instrument (Bio-Rad) and placed into the T100 Thermal Cycler (annealing temperature = 60 °C). Next, the sealed plate was transferred to the QX200™ Droplet Digital™ PCR Systems (Bio-Rad Laboratories) to detect the completed PCR reactions in droplets. We used the QuantaSoft software, version 1.7.4.0917 (Bio-Rad Laboratories), for data analysis. Additional file 1: Table S3 summarises the primer sequences.

## Results

**Fig. 1:**
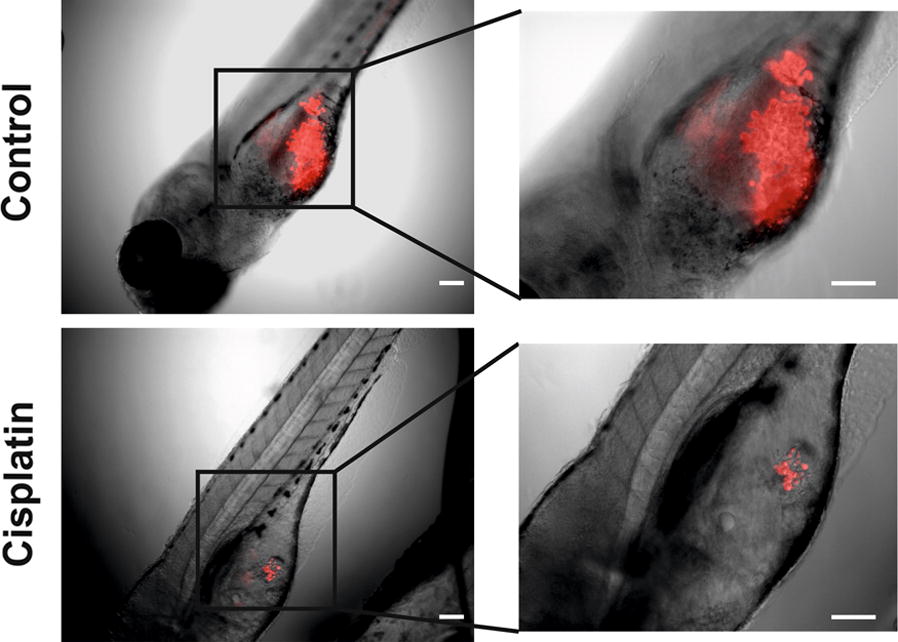
Reduction of the tumour area in zebrafish larvae after cisplatin treatment. Human tongue UT-SCC-24A carcinoma cells were labelled with CellTrace Far Red and injected in the perivitelline space of the zebrafish larvae. For the cisplatin-treated group, cisplatin at a concentration of 3 µg/ml was added to the embryonic medium. Fish were kept at 34 °C for 3 days and then fixed with 4% formaldehyde, mounted on slides and imaged under a microscope. Scale bar = 100 µm

**Fig. 2:**
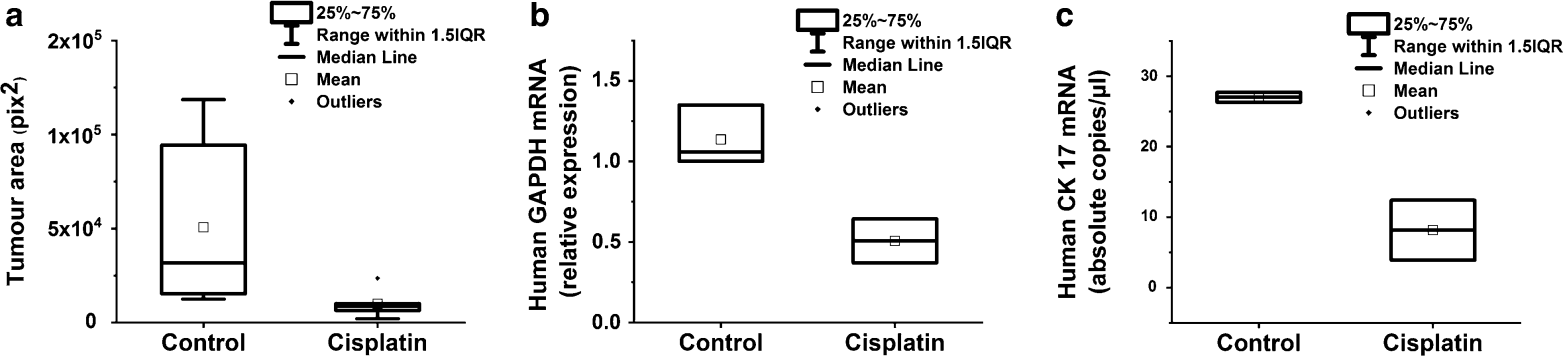
Analysis of the tumour xenograft response in zebrafish larvae to cisplatin treatment using imaging, quantitative and droplet-digital PCR. The evaluation of the cisplatin effect on a human tongue UT-SCC-24A tumour xenograft using imaging technique (**a** 6 fish for each group), quantitative PCR (**b** 20 fish for each group, with 10 fish pooled together) and ddPCR (**c** 20 fish for each group, with 10 fish pooled together). CK 17 = cytokeratin 17

We first compared the already established imaging technique with quantitative PCR and ddPCR in a head-by-head experiment. For quantitative PCR, we used human GAPDH to evaluate the number of human cells in the injected zebrafish larvae. We were unable to use specific epithelial cytokeratin markers in quantitative PCR since the signals fell below the detection levels. Therefore, we employed ddPCR, which successfully detected cytokeratin 17 mRNA. All three techniques showed a clear reduction in the tumour growth in response to cisplatin (Figs. 1, 2). The time required for conducting quantitative PCR of 100 fish was 4.6 h which is around five times less than the imaging assay (21.3 h, Additional file 1: Table S4). Based on these results, we continued the drug testing with quantitative PCR given its convenience and wide availability in most research laboratories.

**Fig. 3:**
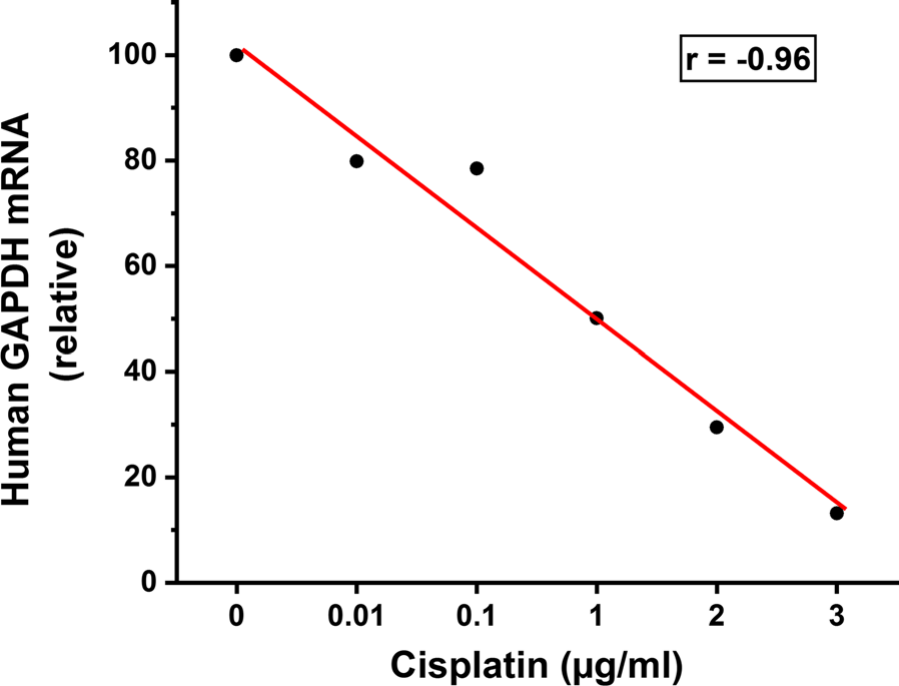
Dose-dependent response of the tumour xenograft to cisplatin treatment evaluated using quantitative PCR. Human larynx carcinoma UT-SCC-42A tumour xenograft in zebrafish larvae was subjected to different concentrations of cisplatin (0–3 µg/ml). The tumour response was evaluated using a quantitative PCR method. Each group had 20 fish, with 10 fish pooled together

Next, we evaluated the dose-dependent response to cisplatin. The curve showed a perfect negative correlation between the cisplatin dose and the human GAPDH signal (r = − 0.96, Fig. 3).

**Fig. 4:**
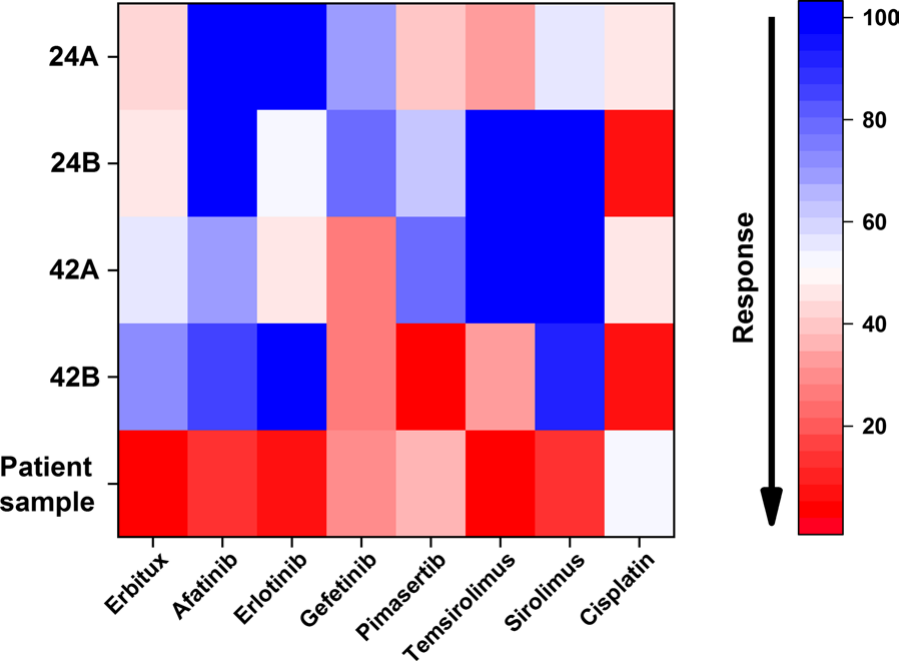
Anti-cancer drug screening against four head and neck squamous cell carcinoma cell lines and one patient-derived tongue carcinoma sample in zebrafish larvae. The heat map represents the response of the cancer cell lines and the patient-derived tongue tumour to eight anti-cancer drugs tested in zebrafish larvae using a quantitative PCR technique. Each group had 20 fish, with 10 fish pooled together. A reduction in the mRNA expression was plotted as a percentage relative to the control (100%)

After these validation procedures for the technique, we initiated drug screening using eight drugs and four cancer cell lines. Cisplatin emerged as the most effective drug in almost all cell lines, while Afatinib showed no activity in any of the cell lines tested (Fig. 4, Additional file 1: Table S5). Other drugs showed different levels of efficacy depending on the tested cell line (Fig. 4, Additional file 1: Table S5).

For the patient-derived (xenograft) tumour cells, cisplatin yielded a modest effect (46% reduction) and the targeted-therapy (EGFR and mTOR inhibitors) revealed a strong effect (80–90% reduction, Fig. 4, Additional file 1: Table S5). Interestingly, all the primary cancer cells (UT-SCC-24A, UT-SCC-42A, and tongue cancer patient sample) were less responsive to cisplatin compared with the metastatic cancer cells (UT-SCC-24B and UT-SCC-42A).

## Discussion

Here, we introduce an easy, fast and low-cost PCR-based in vivo assay to test anti-cancer drug responses in patient-derived tumour samples. The use of zebrafish larvae for drug testing in oncology and for personalised medicine specifically showed promising results, particularly in terms of simulating real patient responses [7, 9]. In this work, we developed the assay further by suggesting the PCR-based technique, which is easier and faster than the imaging assay. The PCR-based method allows for the use of a larger number of fish and tests a larger number of drugs.

We used both quantitative PCR, which is easy and available in almost all research and hospital laboratories, and ddPCR, which is more accurate and could detect mRNA at a very low expression level [16]. For the quantitative PCR, we used human GAPDH, a highly expressed molecule typically used as the housekeeping gene to evaluate the number of human tumour cells. Unfortunately, in quantitative PCR, we could not detect any epithelial cancer cell markers such as cytokeratins, because of their low expression level. For this assay, we used the zebrafish GAPDH as the housekeeping gene, which remained stable between the groups. Using a more sensitive ddPCR method, we detected cytokeratin 17. For patient-derived tumour samples specifically, we found that using ddPCR could be more precise in detecting the signal from epithelial cancer cells alone. Due to the limited availability of ddPCR and since it produced comparable results to quantitative PCR, we continued our assays using the quantitative PCR technique in order to provide an assay which could be easily adopted in most research and clinical facilities.

The dose–response effect shown here and the differing responses to anti-cancer drugs enhance the reliability of the new assay. Additionally, testing patient-derived samples using this assay seems simple, fast and possible using basic laboratory equipment. More importantly, the entire testing procedure for patient samples can be completed within 1 week, which is critical to avoid treatment delays. This possibility may provide preliminary knowledge for the clinician regarding the most suitable and personalised choice of available drugs for a specific patient in a time-efficient manner. In practice, this model appears superior to the mouse xenograft, which takes several weeks to establish drug testing. Additionally, in this system tumour cells are transferred from the patient to the zebrafish larvae within a few hours, without in vitro culturing, which may change their phenotype. Another advantage stems from the need of only 1000 cells per fish, which renders the method feasible even for a small tumour sample.

The primary limitation of the present zebrafish xenograft assay is the different species between the host and the original species (human). This may affect the drug efficacy in some cancers, such as an endocrine-dependent cancer, which should be kept in mind when evaluating the results [17].

## Conclusion

We describe here an improvement to the existing zebrafish larvae xenograft assay applied to in vivo personalised cancer-drug testing. Rather than measuring the tumour size using imaging techniques, we measured the drug efficacy using PCR.

## Additional files

**Additional file 1.** Supplementray tables.

**Additional file 2.** Supplementary video.

## Data Availability

All data and results of this study are available from the corresponding author upon reasonable request.

## Abbreviations

DMEM: Dulbecco’s modified Eagle’s medium
FBS: foetal bovine serum
GAPDH: glyceraldehyde 3-phosphate dehydrogenase
HBSS: Hanks’ Balanced Salt solution
HNSCC: head and neck squamous cell carcinoma
PTU: 1-phenyl 2-thiourea
