# Supplementary material for: PCR-based zebrafish model for personalised medicine in head and neck cancer
Source: J Transl Med. 2019 Jul 22;17:235. doi: 10.1186/s12967-019-1985-1 (PMC6647158; doi:10.1186/s12967-019-1985-1)
Supplement: Supplementary file 1 — Additional file 1. Supplementray tables. [file 12967_2019_1985_MOESM1_ESM.docx]

**Table S1:** Clinical and pathological characteristics of the HNSCC cell lines and the obtained patient sample. TNM is based on pathology report.

| **Cell line** | **Sex^a^** | **Age^b^** | **TNM** | **Specimen site** | **Type^c^** | **Grade** | **Passage** |
| --- | --- | --- | --- | --- | --- | --- | --- |
| **UT-SCC-24A** | M | 41 | T2N0M0 | tongue | pri | G2 | 43 |
| **UT-SCC-24B** | M | 41 | T2N1M0 | neck | met(per) | G2 | 36 |
| **UT-SCC-42A** | M | 43 | T4N3M0 | larynx | pri | G3 | 14 |
| **UT-SCC-42B** | M | 43 | T4N3M0 | neck | met | G3 | 17 |
| **Patient** | M | 70 | T3N3bM0 | Tongue | pri | G2 | NA |
| ^a^M=male , F=female, ^b^ Age in years, ^C^ Pri=primary tumor, met=metastasis, per= persistent disease | | | | | | | |

**Table S2:** List of the anti-cancer compounds used in this study.

| Drug name | Concentration | Supplier |
| --- | --- | --- |
| Cisplatin | 3 µg/ml | Accord Healthcare Ltd. |
| Erbitux | 2.5 µg/ml | Merck KGaA |
| Afatinib | 1 nM | Selleck |
| Erlotinib | 10 nM | Medchem Express |
| Gefitinib | 10 nM | LC Laboratories |
| Pimasertib | 10 nM | Selleck |
| Temsirolimus | 0.1 nM | LC Laboratories |
| Sirolimus | 0.1 nM | LC Laboratories |

**Table S3.** Sequences of the human and zebrafish primers used in this study.

| Species | Gene | Forward | Reverse |
| --- | --- | --- | --- |
| Human | GAPDH | 5’-AAGGTCATCCCTGAGCTG-3’ | 5’-TGCTGTAGCCAAATTCGTTG-3’ |
| Human | CK17 | 5´-ATGTGAAGACGCGGCTGGAGCAGGA-3′ | 5´-ACCTGACGGGTGGTCACCGGTTC-3′ |
| Zebrafish | GAPDH | 5’-AGTGTCAGGACGAACAGAGGCT-3’ | 5’-GCCAATGCGACCGAATCCGTTA-3’ |

**Table S4.** Estimated time required for imaging and PCR based assays.

| Imaging (100 fish) | | PCR (100 fish)^*^ | |
| --- | --- | --- | --- |
| Step | Time (min)^#^ | Step | Time (min)^#^ |
| Sample preparation for fixation | 60 | Fish collection and lysis | 40 |
| Fish fixation (incubation) | 20 | RNA isolation | 60 |
| Fish mounting | 200 | Sample preparation for cDNA synthesis | 30 |
| Imaging | 400 (4 per fish) | cDNA synthesis (incubation) | 26 |
| Image analysis | 600 (6 per fish) | Sample preparation for PCR | 40 |
|  |  | PCR run (incubation) | 48 |
|  |  | Data analysis | 30 |
| Total (without incubation time) | 1260 | Total (without incubation time) | 200 |
| Total | 1280 | Total | 274 |

*Each 10 fish are pooled together in one group.

#The calculated time is based on estimation and it differs from one person to another.

**Table S5.** Anti-cancer drug screening against four head and neck squamous cell carcinoma cell lines and one patient-derived tongue carcinoma sample in zebrafish larvae. Data are presented as mean of human GAPDH expression, mean ΔCT and STD.

**UT-SCC-24A**

| **Drug** | **Mean of human**  **GAPDH expression** | **STD** | **Mean ΔCT** | **STD** |
| --- | --- | --- | --- | --- |
| Control | 0.88 | 0.17 | 11.97 | 0.28 |
| Erbitux | 0.38 | 0.18 | 13.26 | 0.70 |
| Afatinib | 3.16 | 2.93 | 10.52 | 1.60 |
| Erlotinib | 1.01 | 0.31 | 11.80 | 0.46 |
| Gefitinib | 0.60 | 0.18 | 12.56 | 0.45 |
| Pimasertib | 0.35 | 0.24 | 13.47 | 1.08 |
| Temsirolimus | 0.30 | 0.18 | 13.67 | 0.91 |
| Sirolimus | 0.50 | 0.32 | 12.93 | 0.98 |
| Cisplatin | 0.39 | 0.02 | 13.17 | 0.00 |

**UT-SCC-24B**

| **Drug** | **Mean of human**  **GAPDH Expression** | **STD** | **Mean ΔCT** | **STD** |
| --- | --- | --- | --- | --- |
| Control | 1.20 | 0.28 | 13.43 | 0.34 |
| Erbitux | 0.57 | 0.00 | 14.49 | 0.16 |
| Afatinib | 1.42 | 1.80 | 14.34 | 2.97 |
| Erlotinib | 0.62 | 0.63 | 14.90 | 1.87 |
| Gefitinib | 0.94 | 0.91 | 14.22 | 1.71 |
| Pimasertib | 0.74 | 0.28 | 14.16 | 0.56 |
| Temsirolimus | 1.37 | 1.53 | 13.91 | 2.17 |
| Sirolimus | 1.67 | 1.44 | 13.27 | 1.45 |
| Cisplatin | 0.01 | 0.01 | 17.41 | 0.97 |

**UT-SCC-42A**

| **Drug** | **Mean of human**  **GAPDH Expression** | **STD** | **Mean ΔCT** | **STD** |
| --- | --- | --- | --- | --- |
| Control | 1.00 | 0.00 | 11.26 | 0.13 |
| Erbitux | 0.55 | 0.25 | 12.22 | 0.39 |
| Afatinib | 0.69 | 0.28 | 11.87 | 0.60 |
| Erlotinib | 0.47 | 0.15 | 12.39 | 0.46 |
| Gefitinib | 0.27 | 0.17 | 13.29 | 0.94 |
| Pimasertib | 0.80 | 0.63 | 11.85 | 1.28 |
| Temsirolimus | 1.37 | 0.41 | 10.85 | 0.44 |
| Sirolimus | 1.42 | 1.19 | 11.06 | 1.38 |
| Cisplatin | 0.46 | 0.11 | 11.93 | 0.37 |

**UT-SCC-42B**

| **Drug** | **Mean of human**  **GAPDH Expression** | **STD** | **Mean ΔCT** | **STD** |
| --- | --- | --- | --- | --- |
| Control | 1.00 | 0.00 | 8.86 | 0.24 |
| Erbitux | 0.73 | 1.01 | 12.06 | 5.27 |
| Afatinib | 0.87 | 1.19 | 11.23 | 4.45 |
| Erlotinib | 1.45 | 0.00 | 8.32 | 0.13 |
| Gefitinib | 0.28 | 0.12 | 10.77 | 0.62 |
| Pimasertib | 0.04 | 0.00 | 13.52 | 0.36 |
| Temsirolimus | 0.33 | 0.00 | 10.46 | 0.11 |
| Sirolimus | 0.92 | 1.15 | 10.12 | 2.90 |
| Cisplatin | 0.07 | 0.03 | 12.88 | 0.64 |

**Patient sample**

| **Drug** | **Mean of human**  **GAPDH Expression** | **STD** | **Mean ΔCT** | **STD** |
| --- | --- | --- | --- | --- |
| Control | 0.51 | 0.69 | 16.63 | 3.80 |
| Erbitux | 0.01 | 0.01 | 20.42 | 0.73 |
| Afatinib | 0.07 | 0.08 | 18.47 | 2.18 |
| Erlotinib | 0.04 | 0.02 | 18.53 | 0.61 |
| Gefitinib | 0.15 | 0.14 | 17.07 | 1.62 |
| Pimasertib | 0.18 | 0.25 | 18.58 | 4.44 |
| Temsirolimus | 0.02 | 0.00 | 19.57 | 0.00 |
| Sirolimus | 0.08 | 0.01 | 17.65 | 0.14 |
| Cisplatin | 0.28 | 0.00 | 15.80 | 0.01 |
